# Color vision diversity and significance in primates inferred from genetic and field studies

**DOI:** 10.1007/s13258-016-0448-9

**Published:** 2016-07-06

**Authors:** Shoji Kawamura

**Affiliations:** Department of Integrated Biosciences, Graduate School of Frontier Sciences, The University of Tokyo, Bioscience BLDG 502, 5-1-5 Kashiwanoha, Kashiwa, Chiba 277-8562 Japan

**Keywords:** Color vision, Opsin, Primates, New World monkeys

## Abstract

Color provides a reliable cue for object detection and identification during various behaviors such as foraging, mate choice, predator avoidance and navigation. The total number of colors that a visual system can discriminate is largely dependent on the number of different spectral types of cone opsins present in the retina and the spectral separations among them. Thus, opsins provide an excellent model system to study evolutionary interconnections at the genetic, phenotypic and behavioral levels. Primates have evolved a unique ability for three-dimensional color vision (trichromacy) from the two-dimensional color vision (dichromacy) present in the majority of other mammals. This was accomplished via allelic differentiation (e.g. most New World monkeys) or gene duplication (e.g. Old World primates) of the middle to long-wavelength sensitive (M/LWS, or red–green) opsin gene. However, questions remain regarding the behavioral adaptations of primate trichromacy. Allelic differentiation of the M/LWS opsins results in extensive color vision variability in New World monkeys, where trichromats and dichromats are found in the same breeding population, enabling us to directly compare visual performances among different color vision phenotypes. Thus, New World monkeys can serve as an excellent model to understand and evaluate the adaptive significance of primate trichromacy in a behavioral context. I shall summarize recent findings on color vision evolution in primates and introduce our genetic and behavioral study of vision-behavior interrelationships in free-ranging sympatric capuchin and spider monkey populations in Costa Rica.

## A basic knowledge on primate color vision

### Vision specialization of primates

Primates are generally regarded as vision-oriented mammals. The visual system of primates, especially that of anthropoids (simians) [catarrhines (humans, apes and Old World monkeys) and platyrrhines (New World monkeys)], is generally characterized by forward-facing eyes, a postorbital plate (a bony cup surrounding the eye), a fovea (a major central peak in density of cone photoreceptor cells in the retina), and increased representation of the visual centers in the brain cortex (Fleagle 2013). Forward-facing eyes are seen in all primates and enable stereoscopic vision. The postorbital plate is found in simians and prevents the chewing muscles from disrupting eye position, which could serve to improve visual acuity (Heesy et al. 2007). The simian fovea also allows for very high visual acuity. These features are essential for agile movement and saltatory locomotion from branch to branch and appear to have evolved together with other primate pattern traits, such as grasping hands and feet with nails and an opposable thumb/toe, retention of the collar bone allowing for a flexible forelimb movement, and the enlarged brain, for a predominantly arboreal and highly social life (Lambert 1987).

Among features of primate vision, special interest to evolutionary biologists is the unique evolution of trichromatic color vision, which arose from a dichromatic ancestor. Color vision is based on the ability to discriminate light by differences in the wavelength (or hue). At least two different spectral classes of cone photoreceptors are necessary in the retina to perceive differences of wavelength compositions (i.e. colors). Generally speaking, the number of discriminable colors increases as the number of spectrally distinct photoreceptors increases and as the spectral overlap among them is reduced (Vorobyev 2004).

### L/M and S opsins and trichromatic color vision in primates

Vertebrate visual opsins are classified into five phylogenetic types, RH1 (rhodopsin or rod opsin for dim-light vision) and four cone opsins: RH2 (rhodopsin-like, or green), SWS1 (short wavelength-sensitive type 1, or ultraviolet-blue), SWS2 (short wavelength-sensitive type 2, or blue) and M/LWS (middle to long wavelength-sensitive, or red–green) (Yokoyama 2000). After a brief controversy, these five types are now considered to have been present in the common ancestor of all vertebrates including jawless fish (Collin et al. 2003, 2009; Davies et al. 2009a, b, 2012; Pisani et al. 2006; Yokoyama 2000). Thus, early vertebrates could already have had four-dimensional color vision (tetrachromacy). Placental mammals maintain only two types of cone visual opsins, SWS1 and M/LWS, in addition to the RH1 rod opsin, and are hence dichromatic in color vision (Jacobs 1993). Conventionally, in the case of therian mammals, SWS1 opsin is called “blue” or “S” opsin, with λ_max_ at around 410–430 nm among primates, and M/LWS opsins are collectively called “red–green” or “L/M” opsins, with λ_max_ at around 530–560 nm among primates (Kawamura et al. 2012).

Primates are the only exception among placental mammals in attaining trichromatic vision. This is made possible by spectral diversification of the L/M opsin alleles of the single-locus X-linked gene in a few lemuriform primates (Heesy and Ross 2001; Jacobs et al. 2002; Jacobs and Deegan 2003; Tan and Li 1999; Veilleux and Bolnick 2009) and in a majority of New World monkeys (Jacobs 1984, 2007; Matsumoto et al. 2014; Mollon et al. 1984). In this system, all males are dichromatic but females are either dichromatic or trichromatic (“allelic” or “polymorphic” trichromacy). In catarrhine primates and *Alouatta* (howler monkeys, a genus of New World monkeys) (Dulai et al. 1999; Jacobs et al. 1996a; Matsushita et al. 2014), trichromacy was achieved through juxtaposition (duplication) of the spectrally differentiated L/M opsin genes on the same X chromosome (Jacobs and Nathans 2009; Surridge et al. 2003). In this system, both males and females are trichromatic (“routine” trichromacy).

### ‘Three-sites’ rule and variation of primate L/M opsins

The majority of the spectral variation of primate L/M opsin subtypes is explained by amino acid composition at the residue 180, 277 and 285 (‘three-sites’ rule) (Hiramatsu et al. 2004; Matsumoto et al. 2014; Yokoyama and Radlwimmer 1998, 1999, 2001; Yokoyama et al. 2008). The λ_max_ of the L/M opsins with serine, tyrosine and threonine at residues 180, 277 and 285, respectively (denoted SYT), are expected to be approximately 560 nm (Yokoyama et al. 2008). The λ_max_ values of L/M opsins with other three-site combinations can be predicted by subtracting 5, 10 and 17 nm from 560 nm in the case of alanine, phenylalanine and threonine at residues 180, 277 and 285, respectively (Yokoyama et al. 2008). In addition, interactions between these mutations are estimated to be −2 nm for S180A/T285A, +1 nm for Y277F/T285A and +4 nm for S180A/Y277F/T285A (Yokoyama et al. 2008).

**Fig. 1:**
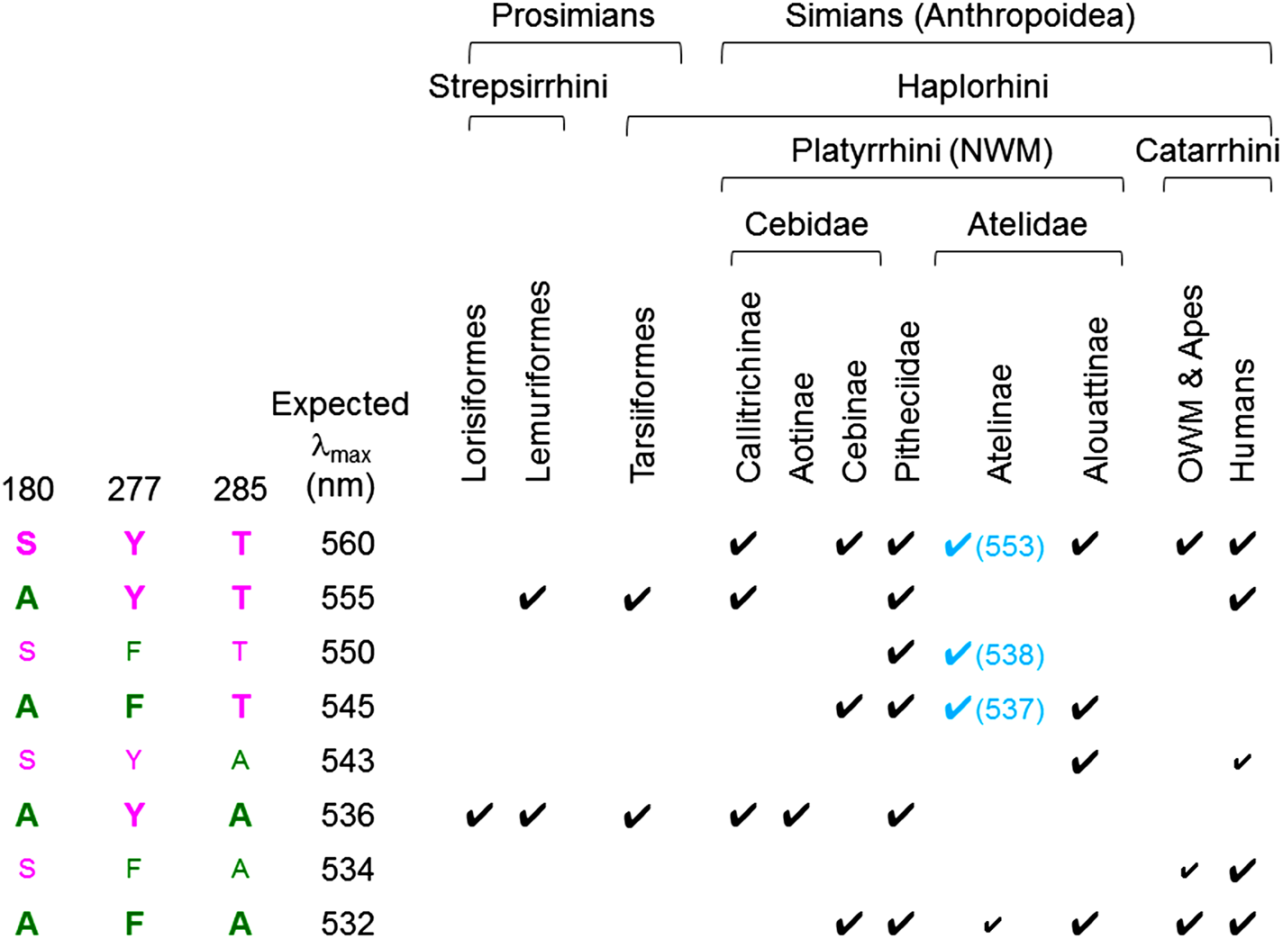
L/M opsin subtypes in primates distinguished on the basis of the ‘three-sites’ composition. At each amino acid site, longer-wave residue (S at 180, Y at 277, T at 285) is indicated with *red* and the shorter-wave residue (A at 180, F at 277, A at 285) is indicated with *green*. The expected λ_max_ values are given to each subtype according to the ‘three-sites’ rule. The major five subtypes found in Platyrrhini are boldfaced and other subtypes are indicated with smaller font. In Lemuriformes, only four species (*Varecia variegata*, *V. rubra*, *Propithecus coquereli*, and *Eulemur macaco flavifrons*) have been reported to retain two subtypes as alleles, while other species examined to date have either one of the two (Heesy and Ross 2001; Jacobs et al. 2002; Jacobs and Deegan 2003; Tan and Li 1999; Veilleux and Bolnick 2009). In Tarsiiformes, extant species have either one of the two subtypes but their common ancestral species is suspected to have had both subtypes as alleles (Melin et al. 2013b; Tan and Li 1999). In Pitheciidae of New World monkeys (NWM), six alleles have been found from bald uakari (*Cacajao calvus*) (Corso et al. 2016). In Atelinae of NWM, λ_max_ values of SYT, SFT and AFT are significantly short-wave shifted (λ_max_ indicated in *parentheses*) from the expectation due to additional mutations and are highlighted with *blue* (Matsumoto et al. 2014). In Atelinae, two alleles are typically found in each species: SYT and SFT in *Ateles*, SYT and AFT in *Lagothrix lagotricha* (Matsumoto et al. 2014) and SYT and SFT in *Brachyteles* (AFA is only found in *Brachyteles hypoxanthus*) (Talebi et al. 2006). In Alouattinae, AFT and SYA are recombinant variants recently reported (Matsushita et al. 2014). In Old World monkeys (OWM) and apes, SFA is reported as a rare recombinant variant in a macaque species *Macaca fascicularis* (Onishi et al. 1999, 2002) and in chimpanzee *Pan troglodytes* (Terao et al. 2005). In humans, a variety of variants are reported (Deeb 2005; Hayashi et al. 2006). AYT is reported as a rare recombinant variant for *Saimiri boliviensis* in Cebinae (Cropp et al. 2002) but omitted here for simplicity

All eight possible combinations of the three sites are reported for primate L/M opsins (Fig. 1). Among them, major five subtypes which spread widely in New World monkeys (SYT, AYT, AFT, AYA, AFA) are estimated to have an antique origin in the common ancestor of New World monkeys (Boissinot et al. 1998). Among the five, AYT and AYA are shared with strepsirhines and tarsiers, and SYT and AFA are shared with catarrhines (Fig. 1). Among the other three, SFT is found only in pithciids (uakari) (Corso et al. 2016) and atelines (spider monkeys, woolly monkeys and muriquis) (Hiramatsu et al. 2005; Matsumoto et al. 2014; Talebi et al. 2006). SYA is found only in howler monkeys as a relatively common recombinant variant (Matsushita et al. 2014) and in humans as a rare recombinant variant (Hayashi et al. 2006) between SYT and AFA. SFA is found only in non-human catarrhines as a rare recombinant variant (Onishi et al. 1999, 2002) and in humans as a common recombinant variant (Deeb 2006) between SYT and AFA. Deviation of observed λ_max_ values from those expected from the ‘three-sites’ rule has been at most 4 nm (Matsushita et al. 2014; Yokoyama et al. 2008). Exceptions to the ‘three-sites’ rule are SYT, SFT and AFT alleles of atelines (Fig. 1), which are short-wave shifted and devoid of the spectral effect of S180A due to unique mutations Y213D and N294K (Matsumoto et al. 2014).

## Preceding machineries enabling primate trichromacy

### X chromosome locality of L/M opsin genes

The therian L/M opsin genes are X-linked, which is extremely important for primates to attain the trichromacy. In the lemuriform primates and New World monkeys with two or more alleles of single-locus L/M opsin genes, these alleles are expressed mutually exclusively by virtue of random X chromosomal inactivation (lyonization) in females and there is only one X chromosome (hemizygosity) in males. Given the uniform presence of the S opsin gene, females having two different spectral alleles of the L/M opsin gene become trichromats, whereas females having two identical L/M opsin alleles and males, due to the hemizygosity of the X chromosome, are dichromats. Thus, the allelic trichromacy is achieved without invention of a special mechanism for allelic exclusion of gene expression.

### Midget ganglion pathway

Among mammals, only primates are equipped with the midget ganglion pathway in the retina, which enables finer resolution of the spectral signals from two spectral classes of L/M cone cells. The midget pathway receives input from only one (in simians) to approximately five (e.g. in bushbabies, a nocturnal lorisiform prosimian) L/M cone cells in the center of a midget ganglion receptive field and compares it to inputs from surrounding L/M cones, allowing high spatial acuity (Martin 1998; Yamada et al. 1998). This neuronal pathway for spatial resolution serves as the evolutionary precursor for the L–M color opponent mechanism, which enables the primate trichromacy instantly when spectral differentiation emerges among L/M cones (Jacobs and Nathans 2009). Although opsin gene renovation appears to confer immediate changes in color perception (Jacobs et al. 2007) without the midget ganglion pathway, evolution of trichromacy may not have been favored in other mammals with coarse spatial resolution even if a similar spectral differentiation of opsin subtypes would have occurred (Surridge et al. 2003; Vorobyev 2004). The absence of trichromatic color vision in other diurnal mammals with similar diets may attest to the acuity requirement of trichromacy (Melin et al. 2014a).

### LCR of juxtaposed L/M opsin genes in catarrhines

In catarrhine primates where L and M opsin genes are juxtaposed on the X chromosome, an additional mechanism is required to selectively express only one gene from the array on one X chromosome. This is achieved through a process of stochastic interaction of a locus control region (LCR), situated upstream of the L/M ospin gene array, with the promoter of only one gene from the array in one cone cell (Smallwood et al. 2002; Wang et al. 1999). The LCR is originally an enhancer element for the single-copy M/LWS opsin gene (Wang et al. 1992). Thus, the gene duplication at the downstream of the LCR enabled catarrhine primates to achieve mutually exclusive expressions of the L and M opsin genes. Hence, primate trichromacy was realized serendipitously through pre-existing situations: X-chromosomal locality of the L/M opsin gene and midget ganglion pathway as well as in catarrhine primates the L/M opsin gene duplication outside the LCR.

In howler monkeys the L and M opsin genes are also juxtaposed (Jacobs et al. 1996a). Unlike catarrhine primates, however, the howlers’ LCR is duplicated together with the opsin gene (Dulai et al. 1999). Nevertheless, mutually exclusive expression of the L and M opsins has been supported by a ganglion recording and microspectrophotometry (MSP) (Silveira et al. 2014). The trichromacy of both male and female howler monkeys is also supported by a behavioral experiment (Araujo et al. 2008). The substantially high peak cone density is observed in the foveal pit of howler monkeys which could be tied with high visual acuity and routine trichromacy (Muniz et al. 2014). These observations imply that howler monkeys have evolved an unknown extra machinery to attain the mutually exclusive expression of the juxtaposed L/M opsin genes and routine trichromacy.

## Visual opsins and the evolutionary origin of primate color vision

### S opsin loss and monochromacy

Variation of S and L/M opsin genes results in various modes of color vision (monochromacy, dichromacy, polymorphism with dichromacy and trichromacy, polymorphic trichromacy, and uniform trichromacy) at various taxonomic levels among primates. The monochromacy (colorblindness) arises in all species of Lorisiformes (lorises and galagos/bushbabies) and *Aotus* (owl monkeys, a genus of New World monkeys) and in various species of cheirogaleid prosimians of Lemuriformes [in genera *Phaner* (fork-marked lemurs), *Cheirogaleus* (dwarf lemurs) and *Allocebus* (hairy-eared mouse lemur)] due to loss of S cone or loss of functional S opsin gene by deleterious mutations (Deegan and Jacobs 1996; Jacobs et al. 1993, 1996b; Kawamura and Kubotera 2004; Levenson et al. 2007; Tan et al. 2005; Veilleux et al. 2013; Wikler and Rakic 1990). These species all share a nocturnal activity pattern.

However, nocturnality does not appear to be a sufficient condition to lose color vision; many other nocturnal primates retain S opsin gene and maintain dichromacy (Jacobs 2013). In fact, the functionality of S opsin gene appears to be maintained by purifying selection in these species (Kawamura and Kubotera 2004; Perry et al. 2007; Tan et al. 2005; Veilleux et al. 2013). Retaining dichromacy in nocturnal strepsirrhines and tarsiers has been suggested as an evidence against the conventional view that ancestral primates were nocturnal, for a hypothesis that the ancestral primates were diurnal or cathemeral and that nocturnality has evolved several times, first in the lorisiforms but much later in other lineages, reflecting different time periods of functional relaxation among lineages (Tan et al. 2005).

### Dichromacy and nocturnality

The diurnal/cathemeral origin hypothesis is losing a support due in part to a recent population genetic study which detected a signature of on-going purifying selection maintaining the S opsin gene in lemuriform nocturnal aye-ayes (*Daubentonia madagascariensis*) (Perry et al. 2007). Aye–ayes have a short-wave shifted S opsin (λ_max_ at 406 nm) (Carvalho et al. 2012) and it is reported that twilight is enriched in short-wavelength (bluish) light with sufficient intensity for aye-ayes with the short-wave-shifted S opsin to perform cone-mediated color vision for their twilight activities (Melin et al. 2012). Another study regards the openness of forest canopy to the sky and the nocturnal activity under moonlight as the main factor influencing the retention of S opsin and color vision in nocturnal lemuriform prosimians (Veilleux et al. 2013). Nocturnal light intensity, particularly short-wave light, is much greater in open canopy forests than in the understory of closed canopy forests (Veilleux and Cummings 2012). Veilleux et al. (2013) found that lemuriform nocturnal species under open canopy habitats generally experience strong purifying selection to maintain the S opsin gene, while, in contrast, those under closed canopy habitats experience weaker purifying selection or a relaxation of selection on it. These studies suggest that dichromatic color vision can be compatible with the nocturnality of ancestral primates.

### Origin of trichromay under dim light

Among strepsirrhines, occasional trichromacy due to the allelic polymorphism of the single-locus X-linked L/M opsin gene has been observed in two diurnal lemurid species [black and white ruffed lemurs (*Varecia variegata*) and red ruffed lemurs (*V. rubra*)], one diurnal indriid species [Coquerel’s sifaka (*Propithecus coquereli*)] and one cathemeral lemurid species [blue-eyed black lemurs (*Eulemur macaco flavifrons*)] (Fig. 1) (Heesy and Ross 2001; Jacobs et al. 2002; Jacobs and Deegan 2003; Tan and Li 1999; Veilleux and Bolnick 2009). Occasional trichromacy is also suspected in the last common ancestor of crown tarsiers (Fig. 1) which is considered to be active in low light, due to the existence of hyper-enlarged eye orbits in the genus (Melin et al. 2013b). With the findings that full moonlight and twilight in tropical forest are sufficient for cone-mediated color vision (Melin et al. 2012), origin of primate trichromay has recently been suggested in activities under dim (mesopic) light conditions (Melin et al. 2013b). Although more data on genetic variation of opsin genes and color vision are necessary for diurnal, cathemeral and nocturnal prosimians, these recent studies challenge the traditional and simplistic view of the diurnal origin of primate trichromacy.

## Visual opsin variation in New World monkeys and evolutionary significance of primate color vision

### Overview and general implications

New World monkeys are known with their extensive inter- and intra-species variation of color vision (Fig. 1). As introduced above, howler monkeys (*Alouatta*) attained the sex-independent trichromacy through juxtaposition of the spectrally differentiated L/M opsin genes on the same X chromosome (Dulai et al. 1999; Jacobs et al. 1996a). On the other extreme, owl monkeys (*Aotus*), the sole nocturnal anthropoid primates, are the cone monochromacy due to the loss of functional S opsin gene (Jacobs et al. 1993, 1996b; Levenson et al. 2007; Wikler and Rakic 1990). All the other 14 genera encompassing all the three platyrrhine families (Cebidae, Atelidae and Pitheciidae) (Wildman et al. 2009) are reported to have allelic polymorphism of the single-locus X-lined L/M opsin gene (Fig. 1) and exhibit color vision variation, i.e. a mixed population of female and male dichromats and female trichromats (Jacobs 2007; Matsumoto et al. 2014).

A wide variation of allelic composition occurs among them (Fig. 1), ranging from two alleles, seen typically in *Ateles* (spider monkeys) and *Lagothrix* (woolly monkeys) (Hiramatsu et al. 2005; Hiwatashi et al. 2010; Jacobs and Deegan 2001; Matsumoto et al. 2014), up to six alleles in *Cacajao calvus* (bald uakari) (Corso et al. 2016). In Pithciidae which *Cacajao* belongs to, similarly many alleles (five alleles) are suspected for *Callicebus moloch* (dusky titi monkeys) by an electroretinogram study (Jacobs and Deegan 2005) although three alleles have been identified in *Callicebus brunneus* (brown titi monkeys) by a nucleotide sequence analysis (Bunce et al. 2011b). Three-allele composition is widely observed in Cebidae (SYT, AFT, AFA in Cebinae, SYT, AYT, AYA in Calitrichinae) (Fig. 1) (de Lima et al. 2015; Jacobs 2007; Matsumoto et al. 2014).

Because of the extensive intra-specific diversity of color vision, New World monkeys are the excellent model to study the utility and evolutionary significance of primate color vision. Different L/M opsin alleles confer different phenotypes on trichromacy and on dichromacy. Generally, larger spectral separation between two L/M opsins in trichromats results in higher red–green chromatic resolution (Melin et al. 2009, 2014b). Likewise, larger spectral separation between S and L/M opsins in dichromats results in higher blue–yellow chromatic resolution (Osorio et al. 2004). Thus, New World monkeys are suited to evaluate the performance difference not only between trichromacy and dichromacy but also between different trichromat phenotypes and between different dichromat phenotypes.

Based on a simplistic view of selective advantage on higher dimension and resolution of color vision in primates, a number of predictions can be made on the variation of L/M opsin subtypes, foraging performance, reproductive success, and so on for different color vision phenotypes in New World monkeys. Recent studies have tested these predictions, with some supported and others not. Emerging is a more complex and condition-dependent nature of utility and evolution of primate trichromatic color vision.

### Unexpected hybrid L/M opsins in howler monkeys

The juxtaposition of L and M ospin genes in howler monkeys was originally reported to consist of the longest (λ_max_ at ~560 nm) and the shortest (λ_max_ at ~530 nm) wave subtypes of L/M opsins in primates, respectively, enabling “normal” and routine trichromacy as seen in catarrhines (Jacobs et al. 1996a). Thus, the finding was taken as a supporting evidence of the evolutionary advantage of trichromacy in primates, regarding polymorphic color vision in most New World monkeys as an intermediate stage of primate evolution from dichromacy to trichromacy with the spectrally most separated L/M opsin subtypes (Bowmaker et al. 1987; Jacobs et al. 1996a).

However, a recent study of natural populations of mantled howlers in Costa Rica and Nicaragua (*Alouatta palliata*) and Yucatan black howlers in Belize (*A. pigra*) found a hybrid L/M opsin gene in each species (“Apa_ML” and “Api_LM”, corresponding to AFT and SYA in Fig. 1, respectively) with ~10 % of frequencies (Matsushita et al. 2014). The λ_max_ of the values of the reconstituted hybrid photopigments are ~546 nm [Matsushita et al. (2014) and our unpublished observation], which should result in mildly “anomalous” trichromats in humans’ term (Deeb 2005) and comparable to those seen in Cebinae carrying an intermediate-λ_max_ allele, who are successful in discriminating stimuli using Ishihara pseudo-isochromatic plates (Saito et al. 2005a). Thus, on the contrary to the prediction, the attained “normal” trichromacy is not maintained in howler monkeys.

### Unequal allele frequencies of L/M opsins

If trichromacy is simply the best in fitness, allele frequencies of the L/M opsin subtypes are expected to be equal to maximize the number of trichromats. In the platyrrhine species with the single-locus L/M opsin alleles and color vision polymorphism, however, accumulated data have proven that this is not the case. Studies on the wild populations of white-faced capuchins (*Cebus capucinus*) and black-handed spider monkeys (*Ateles geoffroyi*) (Hiramatsu et al. 2005; Hiwatashi et al. 2010) and on other spider monkeys (Jacobs and Deegan 2001) and muriquis (Talebi et al. 2006) have found that the longest-wave allele is most frequent and the shortest-wave allele is least frequent. Though with different patterns, unequal allele frequencies are also observed in squirrel monkeys (Cropp et al. 2002) or callitrichines (marmosets and tamarins) (Surridge et al. 2005). Deviation from equality could result from selective neutrality among alleles (Hartl and Clark 2007) and also from a selection not simply maximizing the number of trichromacy. A population genetic study of nucleotide sequence variation revealed that the spectrally differentiated L/M opsin alleles are indeed maintained by selection in the wild populations of capuchin and spider monkeys (Hiwatashi et al. 2010).

The skewed allele frequencies toward longer-wave alleles across the spider monkeys, muriquis and capuchins could imply that the selection consists of complex opposing processes. For trichromats, red–green color discrimination would be greater in individuals having the longest and the shortest wave-sensitive L/M alleles than in individuals having an intermediate wave-sensitive allele. On the other hand, for dichromats, the blue–yellow color resolution would be worst in individuals having the shortest wave-sensitive L/M allele and be best having the longest wave-sensitive allele (Osorio et al. 2004). Thus, the longest wave-sensitive allele would be favored by both trichromats and dichromats, whereas the shortest one would be favored only by trichromats and disfavored by dichromats. Thus, the observed common skew toward longer-wave alleles could indicate that trichromat benefit does not always surpass opposing dichromat benefit and that different alleles could be maintained by different demands among vision types.

However, caution is required to draw a general conclusion because shorter wave alleles can be favored by dichromats in a context to distinguish bluish fruits from background leaves in their long-distance vision (Melin et al. 2014b). Bluish fruits tend to be small and their importance could be larger for small-bodied primates such as squirrel monkeys and callitrichines. This may explain why the longer-wave skewed pattern is not obvious in these species. Shorter wave alleles are also found to be favorable over short distances in computer simulation studies of primate foraging tasks (Melin et al. 2013a; Rowe and Jacobs 2004, 2007), although increased utility of other senses, such as luminance vision and olfaction, could lessen their advantage during short range foraging (Hiramatsu et al. 2008, 2009; Melin et al. 2009).

A complexity is also manifested in the evolutionary history of ateline L/M opsin alleles, which appears to favor thrichromacy on one side but not on the other side. In most atelines, the shortest-wave allele AFA has been lost or is rare (Fig. 1) (Matsumoto et al. 2014), which could imply that dichromat benefit surpasses the opposing trichromat benefit. On the other hand, the spectral separation between the remaining two alleles (SYT and SFT in *Ateles* and *Brachyteles*; SYT and AFT in *Lagothrix*) is enlarged by mutations occurred in the ateline common ancestor, resulting in significant improvement of discriminating conspicuous dietary fruits from leaves in the natural habitat of spider monkeys under both bright and dim light conditions (Matsumoto et al. 2014), which would benefit trichromts. An explanation satisfying both could be that trichromats may tolerate the loss of the shortest-wave allele if the spectral separation of the longest- and intermediate-wave alleles is still sufficient in discriminating stimuli (Saito et al. 2005a) and in foraging performance as shown in capuchin monkeys (Melin et al. 2009). Conversely, in callitrichines, the spectral separation between the longest and the intermediate alleles is comparable to deuteranomalous human trichomats severely impaired in red–green chromatic discrimination (Deeb 2006) (~5 nm; Fig. 1). This may also explain why the shortage of shortest-wave allele is not obvious in callitrichines.

## Behavioral studies and evaluation of trichromacy advantage

### Limited support or contradictive observations for trichromacy advantage

Superior color discrimination abilities of trichromacy are demonstrated in behavioral experiments for captive New World monkeys (Caine et al. 2003; Pessoa et al. 2005; Saito et al. 2005a; Smith et al. 2003b). Finding fruits amid tropical foliage has long been proffered as an adaptive explanation for primate trichromacy. Nevertheless, field observations of free-ranging animals have provided only limited support for simple trichromacy advantage. In a mixed-species troop of saddleback (*Saguinus fuscicollis*) and mustached (*S. mystax*) tamarins, trichromats are further from their neighbors during vigilance than their dichromatic conspecifics. This is explained as resulting from the potentially better perception of predation risk in trichromats (Smith et al. 2005). In a population of white-faced capuchin monkeys (*C. capucinus*), dichromats sniff more figs and take longer foraging sequences than trichromats, especially for cryptic figs, and the trichromat phenotype with the most spectrally separated L/M opsin alleles shows the highest acceptance index for conspicuous figs (Melin et al. 2009). However, there are no differences in feeding rates among phenotypes (Melin et al. 2009).

Results of other behavioral observations of wild New World monkeys have produced equivocal results or results contradictory to the predictions from the trichromat advantage hypothesis. In the wild mixed-species troops of tamarins, the color vision types (dichromatic or trichromatic) do not show a consistent effect on the leadership of the troops to feeding trees (Smith et al. 2003a). In other social groups of tamarins (*S. imperator imperator* and *S. fuscicollis weddelli*) no significant difference is detected between females (thought to consist of trichromats and dichromats) and males (all dichromats) in their ability to locate or discriminate between feeding sites (Dominy et al. 2003a). In a population of capuchin monkeys (*C. capucinus*), no significant difference is detected between trichromats and dichromats in feeding or energy intake rates (Vogel et al. 2007). In another population of the same capuchin monkey species, no difference is detected between dichromats and trichromats in time spent foraging on different food types (Melin et al. 2008). In a free-ranging social group of black-handed spider monkeys, dichromats are not inferior to trichromats in frequency, accuracy, and unit-time intake efficiency of detecting fruits (Hiramatsu et al. 2008). This is explained because the luminance contrast of fruits to background leaves is the main determinant of fruit detection in both dichromats and trichromats on the basis of colorimetric measurement of fruits and background leaves (Hiramatsu et al. 2008). In this social group of spider monkeys, irrespective of color vision phenotypes, the monkeys sniff and reject visually cryptic fruits more often than visually conspicuous fruits, implying that color vision is not the sole determinant for ingestion or rejection of fruits (Hiramatsu et al. 2009).

### Dichromat advantage

Even dichromat advantage is reported in foraging for camouflaged insects in wild capuchins (Melin et al. 2007, 2010) and in wild and captive tamarins (Smith et al. 2012) and in foraging under low light intensity in captive marmosets (Caine et al. 2010). In fact, dichromats are reported to superior to trichromats at breaking camouflage caused by variegated backgrounds (Morgan et al. 1992; Saito et al. 2005b). These findings of observational studies in natural environments suggest that the superior ability of trichromats to see the red–green color contrast may not translate into a selective advantage.

### Direct evaluation of fitness effect of trichromacy

Fedigan et al. (2014) tested whether color vision phenotype is a significant predictor of female fitness in a population of wild capuchins, using 26 years of long-term survival and fertility data. No advantage to trichromats over dichromats for three fitness measures (fertility rates, offspring survival and maternal survival) was found. This finding suggests that a selective mechanism other than simple trichromat advantage (heterozygote advantage) is operating to maintain the color vision polymorphism. More attention should be directed to testing in field studies the alternative mechanisms of balancing selection proposed to explain opsin polymorphism: niche-divergence, frequency-dependence and mutual benefit of association (Fedigan et al. 2014).

### Revising conditions of trichromacy advantage

Recent findings imply that the adaptive value of primate trichromacy is conditional rather than universal, depending on the specific ecological demands on animals in their environments (Kawamura et al. 2012). The question does remain about what exactly these conditions are. Fruits, young leaves, predators and social signals are the main influential visual targets suggested for trichromacy evolution in primates (Allen 1879; Changizi et al. 2006; Dominy and Lucas 2001; Fernandez and Morris 2007; Kamilar et al. 2013; Lucas et al. 1998; Osorio and Vorobyev 1996; Pessoa et al. 2014; Regan et al. 1998, 2001; Sumner and Mollon 2000, 2003; Surridge et al. 2003; Vorobyev 2004). Viewing distance of these objects in tropical foliage is also an important factor in primate color vision (Sumner and Mollon 2000). Although trichromatic color vision is useful for short-range tasks (Parraga et al. 2002), detecting fruits from a distance has long been suggested to confer a more important selective advantage to trichromatic primates (Bompas et al. 2013; Caine 2002; Regan et al. 2001; Sumner and Mollon 2000). However, an observer cannot definitively know when a monkey has detected an object from a distance, and most investigations of primate color vision have been directed to inspection and ingestion behaviors of foods already at close (<2 m) to moderate (<6 m) distances (Caine and Mundy 2000; Hiramatsu et al. 2008; Melin et al. 2009; Smith et al. 2003b; Vogel et al. 2007). Thus, the likely advantage of trichromacy on long-range foraging has been devoted to little attention.

In an effort to address this gap, Melin et al. (2014a, b) reevaluated the trichromacy advantage on fruit foraging of wild capuchin monkeys from a distance by theoretically analyzing computer-simulated conspicuity of fruits from background leaves. In the simulation, trichromatic phenotypes correctly discriminate ca. 70–80 % of the total dietary fruit spectra in a tropical forest. In contrast, less than one-third of the fruits were discriminable to any of dichromatic phenotypes. This general pattern held for the most heavily consumed diet items, preferred foods, or seasonally critical species eaten during periods of overall food dearth. Furthermore, modeled-trichromatic phenotypes are able to discriminate the vast majority of small patch species, anticipated to provide a high finder’s reward. These small resources are suggested to play a critical role in the adaptive value of trichromacy (Bunce et al. 2011a; Melin et al. 2014b).

## Uniform and normal trichromacy in catarrhine primates and exceptional variation in human color vision

### Non-human catarrhines contrasting to platyrrhines

The L and M opsin genes of catarrhine primates are highly similar in nucleotide sequence (~96 % identity) and are closely juxtaposed (Nathans et al. 1986). Thus, they are intrinsically susceptible to recombination and gene conversion between them which could cause hybrid L/M opsin genes, gene loss and gene multiplication (Balding et al. 1992; Deeb et al. 1994; Drummond-Borg et al. 1989; Dulai et al. 1994; Ibbotson et al. 1992; Jorgensen et al. 1990; Shyue et al. 1994; Verrelli and Tishkoff 2004; Winderickx et al. 1992, 1993; Zhao et al. 1998; Zhou and Li 1996). Furthermore, a recombination hot-spot chi element is conserved in the exon 3 among primates (Winderickx et al. 1993). Nevertheless, contrasting to platyrrhine primates, the incidence of color vision variation is remarkably low in non-human catarrhine primates (Jacobs and Williams 2001; Onishi et al. 1999; Terao et al. 2005). Among 744 male long-tailed macaques (*Macaca fascicularis*) examined, only three were found to have a single hybrid L/M opsin gene (SFA in Fig. 1) and to be dichromats (Hanazawa et al. 2001; Onishi et al. 1999). Among 58 male chimpanzees (*Pan troglodytes*), one was found to have a hybrid L/M opsin gene (SFA in Fig. 1) in addition to one normal M opsin gene on the X chromosome and to be an anomalous (protanomalous) trichromat (Saito et al. 2003; Terao et al. 2005). Thus, frequencies of color vision variants in male long-tailed macaques and male chimpanzees can be calculated to be ~0.4 and ~1.7 %, respectively. These frequencies could be overestimated because no variants were found in 455 male monkeys from other macaque species (Onishi et al. 1999) and the chimpanzees examined were from limited numbers of breeding colonies (Terao et al. 2005). Other researchers have reported an absence of color vision defects in Old World monkeys and apes (Hiwatashi et al. 2011; Jacobs and Williams 2001; Verrelli et al. 2008).

Multiple copies of M opsin genes are likely to increase the frequency of unequal recombination events. In humans multiple M copies are found in 66 % of males of European origin (Drummond-Borg et al. 1989) and 56 % of Japanese males (Hayashi et al. 2001). Regarding non-human catarrhines, some studies report that multiple M copies are rare (Onishi et al. 1999, 2002; Terao et al. 2005; Verrelli et al. 2008) yet other studies report that they are common (Dulai et al. 1994; Hiwatashi et al. 2011; Ibbotson et al. 1992). Thus, among Old World monkeys and apes, there seems to be no clear trend on the copy number variation of M opsin gene.

A study of gibbon population samples showed that gene conversion has homogenized L and M opsin genes in introns (Hiwatashi et al. 2011). However, purifying selection against the homogenization has protected the nucleotide difference between L and M opsin genes in centrally located exons, exons 3 and 5 in particular, which include the spectrally crucial ‘three-sites’ (Hiwatashi et al. 2011). This confirms that gene conversions (and perhaps other forms of recombination) do occur between L and M opsin genes in non-human catarrhines but the genes are eliminated from the population by natural selection if gene conversions affect the gene region relevant to spectral difference between L and M opsins. In nonhuman catarrhine primates, even mildly anomalous trichromats have not been found, suggesting a severe selective disadvantage on color vision variants.

The strict conservation of the normal trichromacy in non-human catarrhines is in sharp contrast to New World monkeys. The higher frequency of anomalous trichromacy in New World howler monkeys implies that the selective pressure to maintain “normal” trichromacy is lower in the Neotropics (Matsushita et al. 2014). However, in New World monkey species with polymorphic color vision, genetic studies have shown that the spectrally different alleles of the L/M opsin gene are actively maintained by balancing selection (Boissinot et al. 1998; Hiwatashi et al. 2010; Surridge and Mundy 2002). It is an open question whether the selection is for maintaining (1) simply heterozygotes of L/M opsin alleles, i.e. trichromacy per se; (2) dichromacy and trichromacy; or (3) subtypic variation in dichromacy and/or trichromacy. It is also an open question as to whether the difference between nonhuman catarrhines (uniform and normal trichromacy) and platyrrhines (polymorphic color vision) is attributable to a (1) biogeographic differences among continents, e.g., the severity of seasonality, or a prevalence of drably colored fruits and asynchronous species, e.g., figs and palm fruits (Dominy et al. 2003b); (2) dietary variability, e.g., degree of dependence on insects, leaves, or colorful fruits and different food patch sizes (Melin et al. 2014b); (3) variation in social color signals (Changizi et al. 2006; Fernandez and Morris 2007; Kamilar et al. 2013).

### Uniqueness of humans in color vision

Among catarrhine primates with routine and normal trichromacy, humans constitute a notable exception, in which deletion and multiplication of L/M opsin genes and creation of hybrid L/M opsin genes cause relatively high incidence of dichromacy and anomalous trichromacy, approximately 3–8 % of males being color vision “defects” (Bosten et al. 2005; Deeb 2006; Hood et al. 2006; Sharpe et al. 2006; Verhulst and Maes 1998; Verrelli and Tishkoff 2004). Many humans have multiple copies of the M opsin gene in the L/M opsin gene array where the most upstream gene is typically L and the others are M. Only the upper two genes are expressed and when a hybrid gene occupies either position, it causes anomalous trichromacy (Hayashi et al. 1999). When there is only one L/M opsin gene on an X chromosome or when the two positions are occupied by the same genes, this causes dichromacy (red–green colorblindness: more specifically, protanope when L is lost, and deuteranope when M is lost). These are typically found in men because women have two X chromosomes and thus are more likely to have a “normal” gene array in either one. There are rare cases of individuals, irrespective of sex, who lack functional blue cones (tritanopes, <1:10,000) due to mutations in S opsin gene on chromosome 7 (Sharpe et al. 1999).

The high incidence of color vision variation in humans can be interpreted most conservatively as a result of relaxation of the selective constraint to maintain the spectral difference between the L and M opsin genes. Alternatively, by one step further, adaptive explanations may be possible. Females with a hybrid L/M opsin likely have normal L and M opsins and are able to perceive finer chromatic distinction (Jameson et al. 2001) (a question remains, however, why this is not selected in non-human catarrhines). Studies of New World monkeys provide a profound inference on human color vision variation. The persistence of dichromats in the human population may reflect, as noted above, some advantage in achromatic visual tasks and in having different color vision morphs in a population. Humans are atypical primates, having largely left the foliated environments of forest some million years ago. Then, approximately two million years ago hominins started to devise stone tools and included hunted meat as a considerable portion of their diet. Finally, the increased brain size eventually led to the development of agriculture some thousand years ago and the building of a modern industrial society only a few hundred years ago. The persistence of color vision variation in humans could be related to any of these major events: life outside forest reduces the need for color vision, hunting might have benefited by the presence of dichromatic group members, gathering might have benefited by the presence of hybrid L/M opsins or large agricultural or industrial societies could isolate humans from selection against dichromacy and anomalous trichromacy. It would be crucial to infer when the color vision polymorphism is spread into population as today in human evolution by analyzing L/M opsin nucleotide diversity collected from global ethnic groups.

Finally, color vision is not the sole sense showing diversity among and within species of primates including humans. Interplay of sensory modalities gathers a recent attention in the study of sensory evolution in primates (Hiramatsu et al. 2009).

## References

[CR1] Allen G (1879). The color sense: its origin and development.

[CR2] Araujo AC, Didonet JJ, Araujo CS, Saletti PG, Borges TR, Pessoa VF (2008). Color vision in the black howler monkey (*Alouatta caraya*). Vis Neurosci.

[CR3] Balding DJ, Nichols RA, Hunt DM (1992). Detecting gene conversion: primate visual pigment genes. Proc R Soc B.

[CR4] Boissinot S, Tan Y, Shyue SK, Schneider H, Sampaio I, Neiswanger K, Hewett-Emmett D, Li W-H (1998). Origins and antiquity of X-linked triallelic color vision systems in New World monkeys. Proc Natl Acad Sci USA.

[CR5] Bompas A, Kendall G, Sumner P (2013). Spotting fruit versus picking fruit as the selective advantage of human colour vision. i-Perception.

[CR6] Bosten JM, Robinson JD, Jordan G, Mollon JD (2005). Multidimensional scaling reveals a color dimension unique to ‘color deficient’ observers. Curr Biol.

[CR7] Bowmaker JK, Jacobs GH, Mollon JD (1987). Polymorphism of photopigments in the squirrel monkey: a sixth phenotype. Proc R Soc Lond B.

[CR8] Bunce JA, Isbell LA, Grote MN, Jacobs GH (2011). Color vision variation and foraging behavior in wild neotropical titi monkeys (*Callicebus brunneus*): possible mediating roles for spatial memory and reproductive status. Int J Primatol.

[CR9] Bunce JA, Isbell LA, Neitz M, Bonci D, Surridge AK, Jacobs GH, Smith DG (2011). Characterization of opsin gene alleles affecting color vision in a wild population of titi monkeys (*Callicebus brunneus*). Am J Primatol.

[CR10] Caine NG, Miller LE (2002). Seeing red: consequences of individual differences in color vision in callitrichid primates. Eat or be eaten.

[CR11] Caine NG, Mundy NI (2000). Demonstration of a foraging advantage for trichromatic marmosets (*Callithrix geoffroyi*) dependent on food colour. Proc R Soc Lond B.

[CR12] Caine NG, Surridge AK, Mundy NI (2003). Dichromatic and trichromatic *Callithrix geoffroyi* differ in relative foraging ability for red–green color-camouflaged and non-camouflaged food. Int J Primatol.

[CR13] Caine NG, Osorio D, Mundy NI (2010). A foraging advantage for dichromatic marmosets (*Callithrix geoffroyi*) at low light intensity. Biol Lett.

[CR14] Carvalho LS, Davies WL, Robinson PR, Hunt DM (2012). Spectral tuning and evolution of primate short-wavelength-sensitive visual pigments. Proc R Soc B.

[CR15] Changizi MA, Zhang Q, Shimojo S (2006). Bare skin, blood and the evolution of primate colour vision. Biol Lett.

[CR16] Collin SP, Knight MA, Davies WL, Potter IC, Hunt DM, Trezise AE (2003). Ancient colour vision: multiple opsin genes in the ancestral vertebrates. Curr Biol.

[CR17] Collin SP, Davies WL, Hart NS, Hunt DM (2009). The evolution of early vertebrate photoreceptors. Phil Trans R Soc B.

[CR18] Corso J, Bowler M, Heymann EW, Roos C, Mundy NI (2016). Highly polymorphic colour vision in a New World monkey with red facial skin, the bald uakari (*Cacajao calvus*). Proc R Soc B.

[CR19] Cropp S, Boinski S, Li W-H (2002). Allelic variation in the squirrel monkey X-linked color vision gene: biogeographical and behavioral correlates. J Mol Evol.

[CR20] Davies WL, Carvalho LS, Tay BH, Brenner S, Hunt DM, Venkatesh B (2009). Into the blue: gene duplication and loss underlie color vision adaptations in a deep-sea chimaera, the elephant shark *Callorhinchus milii*. Genome Res.

[CR21] Davies WL, Collin SP, Hunt DM (2009). Adaptive gene loss reflects differences in the visual ecology of basal vertebrates. Mol Biol Evol.

[CR22] Davies WI, Collin SP, Hunt DM (2012). Molecular ecology and adaptation of visual photopigments in craniates. Mol Ecol.

[CR23] de Lima EM, Pessoa DM, Sena L, de Melo AG, de Castro PH, Oliveira-Mendes AC, Schneider MP, Pessoa VF (2015). Polymorphic color vision in captive Uta Hick’s cuxius, or bearded sakis (*Chiropotes utahickae*). Am J Primatol.

[CR24] Deeb SS (2005). The molecular basis of variation in human color vision. Clin Genet.

[CR25] Deeb SS (2006). Genetics of variation in human color vision and the retinal cone mosaic. Curr Opin Genet Dev.

[CR26] Deeb SS, Jorgensen AL, Battisti L, Iwasaki L, Motulsky AG (1994). Sequence divergence of the red and green visual pigments in great apes and humans. Proc Natl Acad Sci USA.

[CR27] Deegan JF, Jacobs GH (1996). Spectral sensitivity and photopigments of a nocturnal prosimian, the bushbaby (*Otolemur crassicaudatus*). Am J Primatol.

[CR28] Dominy NJ, Lucas PW (2001). Ecological importance of trichromatic vision to primates. Nature.

[CR29] Dominy NJ, Garber PA, Bicca-Marques JC, Azevedo-Lopes MA (2003). Do female tamarins use visual cues to detect fruit rewards more successfully than do males?. Anim Behav.

[CR30] Dominy NJ, Svenning JC, Li W-H (2003). Historical contingency in the evolution of primate color vision. J Hum Evol.

[CR31] Drummond-Borg M, Deeb SS, Motulsky AG (1989). Molecular patterns of X chromosome-linked color vision genes among 134 men of European ancestry. Proc Natl Acad Sci USA.

[CR32] Dulai KS, Bowmaker JK, Mollon JD, Hunt DM (1994). Sequence divergence, polymorphism and evolution of the middle-wave and long-wave visual pigment genes of great apes and Old World monkeys. Vision Res.

[CR33] Dulai KS, von Dornum M, Mollon JD, Hunt DM (1999). The evolution of trichromatic color vision by opsin gene duplication in New World and Old World primates. Genome Res.

[CR34] Fedigan LM, Melin AD, Addicott JF, Kawamura S (2014). The heterozygote superiority hypothesis for polymorphic color vision is not supported by long-term fitness data from wild neotropical monkeys. PLoS ONE.

[CR35] Fernandez AA, Morris MR (2007). Sexual selection and trichromatic color vision in primates: statistical support for the preexisting-bias hypothesis. Am Nat.

[CR36] Fleagle JG (2013). Primate adaptation and evolution.

[CR37] Hanazawa A, Mikami A, Sulistyo Angelika P, Takenaka O, Goto S, Onishi A, Koike S, Yamamori T, Kato K, Kondo A, Suryobroto B, Farajallah A, Komatsu H (2001). Electroretinogram analysis of relative spectral sensitivity in genetically identified dichromatic macaques. Proc Natl Acad Sci USA.

[CR38] Hartl DL, Clark AG (2007). Principles of population genetics.

[CR39] Hayashi T, Motulsky AG, Deeb SS (1999). Position of a ‘green–red’ hybrid gene in the visual pigment array determines colour-vision phenotype. Nat Genet.

[CR40] Hayashi S, Ueyama H, Tanabe S, Yamade S, Kani K (2001). Number and variations of the red and green visual pigment genes in Japanese men with normal color vision. Jpn J Ophthalmol.

[CR41] Hayashi T, Kubo A, Takeuchi T, Gekka T, Goto-Omoto S, Kitahara K (2006). Novel form of a single X-linked visual pigment gene in a unique dichromatic color-vision defect. Vis Neurosci.

[CR42] Heesy CP, Ross CF (2001). Evolution of activity patterns and chromatic vision in primates: morphometrics, genetics and cladistics. J Hum Evol.

[CR43] Heesy CP, Ross CF, Demes B, Ravosa MJ, Dagosto M (2007). Oculomotor stability and the functions of the postorbital bar and septum. Primate origins: adaptations and evolution.

[CR44] Hiramatsu C, Radlwimmer FB, Yokoyama S, Kawamura S (2004). Mutagenesis and reconstitution of middle-to-long-wave-sensitive visual pigments of New World monkeys for testing the tuning effect of residues at sites 229 and 233. Vision Res.

[CR45] Hiramatsu C, Tsutsui T, Matsumoto Y, Aureli F, Fedigan LM, Kawamura S (2005). Color-vision polymorphism in wild capuchins (*Cebus capucinus*) and spider monkeys (*Ateles geoffroyi*) in Costa Rica. Am J Primatol.

[CR46] Hiramatsu C, Melin AD, Aureli F, Schaffner CM, Vorobyev M, Matsumoto Y, Kawamura S (2008). Importance of achromatic contrast in short-range fruit foraging of primates. PLoS ONE.

[CR47] Hiramatsu C, Melin AD, Aureli F, Schaffner CM, Vorobyev M, Kawamura S (2009). Interplay of olfaction and vision in fruit foraging of spider monkeys. Anim Behav.

[CR48] Hiwatashi T, Okabe Y, Tsutsui T, Hiramatsu C, Melin AD, Oota H, Schaffner CM, Aureli F, Fedigan LM, Innan H, Kawamura S (2010). An explicit signature of balancing selection for color-vision variation in New World monkeys. Mol Biol Evol.

[CR49] Hiwatashi T, Mikami A, Katsumura T, Suryobroto B, Perwitasari-Farajallah D, Malaivijitnond S, Siriaroonrat B, Oota H, Goto S, Kawamura S (2011). Gene conversion and purifying selection shape nucleotide variation in gibbon L/M opsin genes. BMC Evol Biol.

[CR50] Hood SM, Mollon JD, Purves L, Jordan G (2006). Color discrimination in carriers of color deficiency. Vision Res.

[CR51] Ibbotson RE, Hunt DM, Bowmaker JK, Mollon JD (1992). Sequence divergence and copy number of the middle- and long-wave photopigment genes in Old World monkeys. Proc R Soc Lond B.

[CR52] Jacobs GH (1984). Within-species variations in visual capacity among squirrel monkeys (*Saimiri sciureus*): color vision. Vision Res.

[CR53] Jacobs GH (1993). The distribution and nature of colour vision among the mammals. Biol Rev.

[CR54] Jacobs GH (2007). New World monkeys and color. Int J Primatol.

[CR55] Jacobs GH (2013). Losses of functional opsin genes, short-wavelength cone photopigments, and color vision-A significant trend in the evolution of mammalian vision. Vis Neurosci.

[CR56] Jacobs GH, Deegan JF (2001). Photopigments and colour vision in New World monkeys from the family Atelidae. Proc R Soc Lond B.

[CR57] Jacobs GH, Deegan JF, Mollon JD, Pokorny J, Knoblanch K (2003). Photopigment polymorphism in prosimians and the origins of primate trichromacy. Normal and defective colour vision.

[CR58] Jacobs GH, Deegan JF (2005). Polymorphic New World monkeys with more than three M/L cone types. J Opt Soc Am A:.

[CR59] Jacobs GH, Nathans J (2009). The evolution of primate color vision. Sci Am.

[CR60] Jacobs GH, Williams GA (2001). The prevalence of defective color vision in Old World monkeys and apes. Col Res Appl.

[CR61] Jacobs GH, Deegan JF, Neitz J, Crognale MA, Neitz M (1993). Photopigments and color vision in the nocturnal monkey, *Aotus*. Vision Res.

[CR62] Jacobs GH, Neitz M, Deegan JF, Neitz J (1996). Trichromatic colour vision in New World monkeys. Nature.

[CR63] Jacobs GH, Neitz M, Neitz J (1996). Mutations in S-cone pigment genes and the absence of colour vision in two species of nocturnal primate. Proc R Soc Lond B.

[CR64] Jacobs GH, Deegan JF, Tan Y, Li W-H (2002). Opsin gene and photopigment polymorphism in a prosimian primate. Vision Res.

[CR65] Jacobs GH, Williams GA, Cahill H, Nathans J (2007). Emergence of novel color vision in mice engineered to express a human cone photopigment. Science.

[CR66] Jameson KA, Highnote SM, Wasserman LM (2001). Richer color experience in observers with multiple photopigment opsin genes. Psychon Bull Rev.

[CR67] Jorgensen AL, Deeb SS, Motulsky AG (1990). Molecular genetics of X chromosome-linked color vision among populations of African and Japanese ancestry: high frequency of a shortened red pigment gene among Afro–Americans. Proc Natl Acad Sci USA.

[CR68] Kamilar JM, Heesy CP, Bradley BJ (2013). Did trichromatic color vision and red hair color coevolve in primates?. Am J Primatol.

[CR69] Kawamura S, Kubotera N (2004). Ancestral loss of short wave-sensitive cone visual pigment in lorisiform prosimians, contrasting with its strict conservation in other prosimians. J Mol Evol.

[CR70] Kawamura S, Hiramatsu C, Melin AD, Schaffner CM, Aureli F, Fedigan LM, Hirai H, Imai H, Go Y (2012). Polymorphic color vision in primates: evolutionary considerations. Post-genome biology of primates.

[CR71] Lambert D (1987). The cambridge guide to prehistoric man.

[CR72] Levenson DH, Fernandez-Duque E, Evans S, Jacobs GH (2007). Mutational changes in S-cone opsin genes common to both nocturnal and cathemeral *Aotus* monkeys. Am J Primatol.

[CR73] Lucas PW, Darvell BW, Lee PKD, Yuen TDB, Choong MF (1998). Colour cues for leaf food selection by long-tailed macaques (*Macaca fascicularis*) with a new suggestion for the evolution of trichromatic colour vision. Folia Primatol.

[CR74] Martin PR (1998). Colour processing in the primate retina: recent progress. J Physiol.

[CR75] Matsumoto Y, Hiramatsu C, Matsushita Y, Ozawa N, Ashino R, Nakata M, Kasagi S, Di Fiore A, Schaffner CM, Aureli F, Melin AD, Kawamura S (2014). Evolutionary renovation of L/M opsin polymorphism confers a fruit discrimination advantage to ateline New World monkeys. Mol Ecol.

[CR76] Matsushita Y, Oota H, Welker BJ, Pavelka MS, Kawamura S (2014). Color vision variation as evidenced by hybrid L/M opsin genes in wild populations of trichromatic *Alouatta* New World monkeys. Int J Primatol.

[CR77] Melin AD, Fedigan LM, Hiramatsu C, Sendall CL, Kawamura S (2007). Effects of colour vision phenotype on insect capture by a free-ranging population of white-faced capuchins (*Cebus capucinus*). Anim Behav.

[CR78] Melin AD, Fedigan LM, Hiramatsu C, Kawamura S (2008). Polymorphic color vision in white-faced capuchins (*Cebus capucinus*): is there foraging niche divergence among phenotypes?. Behav Ecol Sociobiol.

[CR79] Melin AD, Fedigan LM, Hiramatsu C, Hiwatashi T, Parr N, Kawamura S (2009). Fig foraging by dichromatic and trichromatic *Cebus capucinus* in a tropical dry forest. Int J Primatol.

[CR80] Melin AD, Fedigan LM, Young HC, Kawamura S (2010). Can color vision variation explain sex differences in invertebrate foraging by capuchin monkeys?. Curr Zool.

[CR81] Melin AD, Moritz GL, Fosbury RAE, Kawamura S, Dominy NJ (2012). Why aye–ayes see blue. Am J Primatol.

[CR82] Melin AD, Kline DW, Hickey C, Fedigan LM (2013). Food search through the eyes of a monkey: a functional substitution approach for assessing the ecology of primate color vision. Vision Res.

[CR83] Melin AD, Matsushita Y, Moritz GL, Dominy NJ, Kawamura S (2013). Inferred L/M cone opsin polymorphism of ancestral tarsiers sheds dim light on the origin of anthropoid primates. Proc R Soc B.

[CR84] Melin AD, Danosi CF, McCracken GF, Dominy NJ (2014). Dichromatic vision in a fruit bat with diurnal proclivities: the Samoan flying fox (*Pteropus samoensis*). J Comp Physiol A.

[CR85] Melin AD, Hiramatsu C, Parr NA, Matsushita Y, Kawamura S, Fedigan LM (2014). The behavioral ecology of color vision: considering fruit conspicuity, detection distance and dietary importance. Int J Primatol.

[CR86] Mollon JD, Bowmaker JK, Jacobs GH (1984). Variations of colour vision in a New World primate can be explained by polymorphism of retinal photopigments. Proc R Soc Lond B.

[CR87] Morgan MJ, Adam A, Mollon JD (1992). Dichromats detect colour-camouflaged objects that are not detected by trichromats. Proc R Soc Lond B.

[CR88] Muniz JAPC, de Athaide LM, Gomes BD, Finlay BL, de Lima Silveira LC (2014). Ganglion cell and displaced amacrine cell density distribution in the retina of the howler monkey (*Alouatta caraya*). PLoS ONE.

[CR89] Nathans J, Thomas D, Hogness DS (1986). Molecular genetics of human color vision: the genes encoding blue, green, and red pigments. Science.

[CR90] Onishi A, Koike S, Ida M, Imai H, Shichida Y, Takenaka O, Hanazawa A, Komatsu H, Mikami A, Goto S, Suryobroto B, Kitahara K, Yamamori T, Konatsu H (1999). Dichromatism in macaque monkeys. Nature.

[CR91] Onishi A, Koike S, Ida-Hosonuma M, Imai H, Shichida Y, Takenaka O, Hanazawa A, Komatsu H, Mikami A, Goto S, Suryobroto B, Farajallah A, Varavudhi P, Eakavhibata C, Kitahara K, Yamamori T (2002). Variations in long- and middle-wavelength-sensitive opsin gene loci in crab-eating monkeys. Vision Res.

[CR92] Osorio D, Vorobyev M (1996). Colour vision as an adaptation to frugivory in primates. Proc R Soc Lond B.

[CR93] Osorio D, Smith AC, Vorobyev M, Buchanan-Smith HM (2004). Detection of fruit and the selection of primate visual pigments for color vision. Am Nat.

[CR94] Parraga CA, Troscianko T, Tolhurst DJ (2002). Spatiochromatic properties of natural images and human vision. Curr Biol.

[CR95] Perry GH, Martin RD, Verrelli BC (2007). Signatures of functional constraint at aye-aye opsin genes: the potential of adaptive color vision in a nocturnal primate. Mol Biol Evol.

[CR96] Pessoa DM, Tomaz C, Pessoa VF (2005). Color vision in marmosets and tamarins: behavioral evidence. Am J Primatol.

[CR97] Pessoa DM, Maia R, de Albuquerque Ajuz RC, De Moraes PZ, Spyrides MH, Pessoa VF (2014). The adaptive value of primate color vision for predator detection. Am J Primatol.

[CR98] Pisani D, Mohun SM, Harris SR, McInerney JO, Wilkinson M (2006). Molecular evidence for dim-light vision in the last common ancestor of the vertebrates. Curr Biol.

[CR99] Regan BC, Julliot C, Simmen B, Vienot F, Charles-Dominique P, Mollon JD (1998). Frugivory and colour vision in *Alouatta seniculus*, a trichromatic platyrrhine monkey. Vis Res.

[CR100] Regan BC, Julliot C, Simmen B, Vienot F, Charles-Dominique P, Mollon JD (2001). Fruits, foliage and the evolution of primate colour vision. Phil Trans R Soc B.

[CR101] Rowe MP, Jacobs GH (2004). Cone pigment polymorphism in New World monkeys: are all pigments created equal?. Vis Neurosci.

[CR102] Rowe MP, Jacobs GH (2007). Naturalistic color discriminations in polymorphic platyrrhine monkeys: effects of stimulus luminance and duration examined with functional substitution. Vis Neurosci.

[CR103] Saito A, Mikami A, Hasegawa T, Koida K, Terao K, Koike S, Onishi A, Takenaka O, Teramoto M, Mori Y (2003). Behavioral evidence of color vision deficiency in a protanomalia chimpanzee (*Pan troglodytes*). Primates.

[CR104] Saito A, Kawamura S, Mikami A, Ueno Y, Hiramatsu C, Koida K, Fujita K, Kuroshima H, Hasegawa T (2005). Demonstration of a genotype-phenotype correlation in the polymorphic color vision of a non-callitrichine New World monkey, capuchin (*Cebus apella*). Am J Primatol.

[CR105] Saito A, Mikami A, Kawamura S, Ueno Y, Hiramatsu C, Widayati KA, Suryobroto B, Teramoto M, Mori Y, Nagano K, Fujita K, Kuroshima H, Hasegawa T (2005). Advantage of dichromats over trichromats in discrimination of color-camouflaged stimuli in nonhuman primates. Am J Primatol.

[CR106] Sharpe LT, Stockman A, Jagle H, Nathans J, Gegenfurtner KR, Sharpe LT (1999). Opsin genes, cone photopigments, color vision, and color blindness. Color vision: from genes to perception.

[CR107] Sharpe LT, de Luca E, Hansen T, Jagle H, Gegenfurtner KR (2006). Advantages and disadvantages of human dichromacy. J Vis.

[CR108] Shyue SK, Li L, Chang BH, Li W-H (1994). Intronic gene conversion in the evolution of human X-linked color vision genes. Mol Biol Evol.

[CR109] Silveira LCL, Saito CA, da Silva Filho M, Kremers J, Bowmaker JK, Lee BB (2014). *Alouatta* trichromatic color vision: cone spectra and physiological responses studied with microspectrophotometry and single unit retinal electrophysiology. PLoS ONE.

[CR110] Smallwood PM, Wang Y, Nathans J (2002). Role of a locus control region in the mutually exclusive expression of human red and green cone pigment genes. Proc Natl Acad Sci USA.

[CR111] Smith AC, Buchanan-Smith HM, Surridge AK, Mundy NI (2003). Leaders of progressions in wild mixed-species troops of saddleback (*Saguinus fuscicollis*) and mustached tamarins (*S. mystax*), with emphasis on color vision and sex. Am J Primatol.

[CR112] Smith AC, Buchanan-Smith HM, Surridge AK, Osorio D, Mundy NI (2003). The effect of colour vision status on the detection and selection of fruits by tamarins (*Saguinus* spp.). J Exp Biol.

[CR113] Smith AC, Buchanan-Smith HM, Surridge AK, Mundy NI (2005). Factors affecting group spread within wild mixed-species troops of saddleback and mustached tamarins. Int J Primatol.

[CR114] Smith AC, Surridge AK, Prescott MJ, Osorio D, Mundy NI, Buchanan-Smith HM (2012). Effect of colour vision status on insect prey capture efficiency of captive and wild tamarins (*Saguinus* spp.). Anim Behav.

[CR115] Sumner P, Mollon JD (2000). Catarrhine photopigments are optimized for detecting targets against a foliage background. J Exp Biol.

[CR116] Sumner P, Mollon JD (2003). Colors of primate pelage and skin: objective assessment of conspicuousness. Am J Primatol.

[CR117] Surridge AK, Mundy NI (2002). Trans-specific evolution of opsin alleles and the maintenance of trichromatic colour vision in Callitrichine primates. Mol Ecol.

[CR118] Surridge AK, Osorio D, Mundy NI (2003). Evolution and selection of trichromatic vision in primates. Trends Ecol Evol.

[CR119] Surridge AK, Suarez SS, Buchanan-Smith HM, Smith AC, Mundy NI (2005). Color vision pigment frequencies in wild tamarins (*Saguinus* spp.). Am J Primatol.

[CR120] Talebi MG, Pope TR, Vogel ER, Neitz M, Dominy NJ (2006). Polymorphism of visual pigment genes in the muriqui (Primates, Atelidae). Mol Ecol.

[CR121] Tan Y, Li W-H (1999). Trichromatic vision in prosimians. Nature.

[CR122] Tan Y, Yoder AD, Yamashita N, Li W-H (2005). Evidence from opsin genes rejects nocturnality in ancestral primates. Proc Natl Acad Sci USA.

[CR123] Terao K, Mikami A, Saito A, Itoh S, Ogawa H, Takenaka O, Sakai T, Onishi A, Teramoto M, Udono T, Emi Y, Kobayashi H, Imai H, Shichida Y, Koike S (2005). Identification of a protanomalous chimpanzee by molecular genetic and electroretinogram analyses. Vision Res.

[CR124] Veilleux CC, Bolnick DA (2009). Opsin gene polymorphism predicts trichromacy in a cathemeral lemur. Am J Primatol.

[CR125] Veilleux CC, Cummings ME (2012). Nocturnal light environments and species ecology: implications for nocturnal color vision in forests. J Exp Biol.

[CR126] Veilleux CC, Louis EE, Bolnick DA (2013). Nocturnal light environments influence color vision and signatures of selection on the *OPN1SW* opsin gene in nocturnal lemurs. Mol Biol Evol.

[CR127] Verhulst S, Maes FW (1998). Scotopic vision in colour-blinds. Vision Res.

[CR128] Verrelli BC, Tishkoff SA (2004). Signatures of selection and gene conversion associated with human color vision variation. Am J Hum Genet.

[CR129] Verrelli BC, Lewis CM, Stone AC, Perry GH (2008). Different selective pressures shape the molecular evolution of color vision in chimpanzee and human populations. Mol Biol Evol.

[CR130] Vogel ER, Neitz M, Dominy NJ (2007). Effect of color vision phenotype on the foraging of wild white-faced capuchins, *Cebus capucinus*. Behav Ecol.

[CR131] Vorobyev M (2004). Ecology and evolution of primate colour vision. Clin Exp Optom.

[CR132] Wang Y, Macke JP, Merbs SL, Zack DJ, Klaunberg B, Bennett J, Gearhart J, Nathans J (1992). A locus control region adjacent to the human red and green visual pigment genes. Neuron.

[CR133] Wang Y, Smallwood PM, Cowan M, Blesh D, Lawler A, Nathans J (1999). Mutually exclusive expression of human red and green visual pigment-reporter transgenes occurs at high frequency in murine cone photoreceptors. Proc Natl Acad Sci USA.

[CR134] Wikler KC, Rakic P (1990). Distribution of photoreceptor subtypes in the retina of diurnal and nocturnal primates. J Neurosci.

[CR135] Wildman DE, Jameson NM, Opazo JC, Yi SV (2009). A fully resolved genus level phylogeny of neotropical primates (Platyrrhini). Mol Phylogenet Evol.

[CR136] Winderickx J, Lindsey DT, Sanocki E, Teller DY, Motulsky AG, Deeb SS (1992). Polymorphism in red photopigment underlies variation in colour matching. Nature.

[CR137] Winderickx J, Battisti L, Hibiya Y, Motulsky AG, Deeb SS (1993). Haplotype diversity in the human red and green opsin genes: evidence for frequent sequence exchange in exon 3. Hum Mol Genet.

[CR138] Yamada ES, Marshak DW, Silveira LC, Casagrande VA (1998). Morphology of P and M retinal ganglion cells of the bush baby. Vision Res.

[CR139] Yokoyama S (2000). Molecular evolution of vertebrate visual pigments. Prog Retin Eye Res.

[CR140] Yokoyama S, Radlwimmer FB (1998). The “five-sites” rule and the evolution of red and green color vision in mammals. Mol Biol Evol.

[CR141] Yokoyama S, Radlwimmer FB (1999). The molecular genetics of red and green color vision in mammals. Genetics.

[CR142] Yokoyama S, Radlwimmer FB (2001). The molecular genetics and evolution of red and green color vision in vertebrates. Genetics.

[CR143] Yokoyama S, Yang H, Starmer WT (2008). Molecular basis of spectral tuning in the red- and green-sensitive (M/LWS) pigments in vertebrates. Genetics.

[CR144] Zhao Z, Hewett-Emmett D, Li W-H (1998). Frequent gene conversion between human red and green opsin genes. J Mol Evol.

[CR145] Zhou YH, Li W-H (1996). Gene conversion and natural selection in the evolution of X-linked color vision genes in higher primates. Mol Biol Evol.

